# The Effect of COVID-19 on Type 1 Diabetes Occurrence among Children and Adolescents: A Multicenter Prospective Observational Cohort Study in Israel

**DOI:** 10.1155/2023/6659719

**Published:** 2023-11-14

**Authors:** Noah Gruber, Liat Brand, Ehud Barhod, Rina Hemi, Yael Lebenthal, Marianna Rachmiel, Tal Kedar, Rachel Shatzman-Steuerman, Rachael Sverdlove, Yaniv Lustig, Victoria Indenbaum, Orit Pinhas-Hamiel

**Affiliations:** ^1^Pediatric Endocrine and Diabetes Unit, Edmond and Lily Safra Children's Hospital, Sheba Medical Center, Ramat Gan, Israel; ^2^Sackler School of Medicine, Tel-Aviv University, Tel Aviv-Yafo, Israel; ^3^The Dalia and David Arabov Diabetes Research Center, Division of Endocrinology, Diabetes and Metabolism, Sheba Medical Center, Tel HaShomer, Israel; ^4^Pediatric Endocrinology and Diabetes Unit, Dana-Dwek Children's Hospital, Tel Aviv Sourasky Medical Center, Tel Aviv, Israel; ^5^Pediatric Endocrinology and Diabetes Institute, Shamir Medical Center (Assaf Harofeh), Be'er Ya'akov, Israel; ^6^Pediatric Infectious Unit, Edmond and Lily Safra Children's Hospital, Sheba Medical Center, Ramat Gan, Israel; ^7^Central Virology Laboratory, Ministry of Health, Sheba Medical Center, Tel HaShomer, Israel

## Abstract

**Aim:**

The effect of severe acute respiratory syndrome coronavirus 2 (SARS-CoV-2) infection on the pediatric occurrence of type 1 diabetes (T1D) is inconclusive. We aimed to assess associations between seroprevalences of the distinct anti-SARS-CoV-2 antibodies and T1D occurrence in children and adolescents.

**Methods:**

This multicenter prospective observational cohort comprised children diagnosed with T1D between October 2020 and July 2022 and unrelated children who performed endocrine tests (control group) in a 1 : 3 ratio. Anti-SARS-CoV-2 antibodies, including anti-S, anti-N, and neutralizing antibodies, were assessed in each group.

**Results:**

The cohort included 51 children with T1D and 182 children in the control group. The median (interquartile range) age was 11.4 (8.2, 13.3) years, with 45% being female. Increases were not observed in the seroprevalence of any of the anti-SARS-CoV-2 antibodies among the children with new-onset T1D compared to the control group. Among the T1D group, anti-S seroprevalence was higher among those without diabetic ketoacidosis (DKA) than in those with DKA upon T1D diagnosis (72% vs. 42%, *p*=0.035). After adjustment to vaccination status, this difference was not statistically significant. Additionally, anti-N antibodies and neutralizing antibodies did not differ between the DKA and the non-DKA groups. None of the anti-SARS-CoV-2 antibodies were associated with any of the glycemic parameters.

**Conclusions:**

This study is the first to assess several distinct anti-SARS-CoV-2 antibodies in new-onset T1D, and our findings do not support an association between SARS-CoV-2 infection and the occurrence of T1D in children and adolescents. Since autoimmunity may emerge years after a viral infection, we recommend conducting follow-up epidemiological studies to assess whether there is a change in the incidence of T1D following the SARS-CoV-2 pandemic.

## 1. Introduction

The incidence of type 1 diabetes (T1D), one of the most common chronic diseases in children, is increasing worldwide [1]. T1D is a complex autoimmune disorder characterized by the selective destruction of pancreatic *β*-cells. The precise etiology of T1D is still unknown, but viruses have long been suggested as potential environmental triggers for the disease [1].

In February 2020, the World Health Organization declared the severe acute respiratory syndrome coronavirus 2 (SARS-CoV-2) and the disease caused by it, coronavirus disease 2019 (COVID-19), as a global pandemic. In Israel, the pandemic also began in February 2020, with peaks of new diagnoses in September 2020, January 2020, September 2021, January 2022, March 2022, and July 2022 [2]. Individuals above 18 years of age began receiving the vaccine against SARS-CoV-2 (namely, the BNT162b2 COVID-19 vaccine) in December 2020, children aged 12–18 years in July 2021, and children aged 5–12 years in December 2021 [3, 4].

There is evidence of a postautoimmune process after COVID-19 [5, 6]. In addition, the presence of SARS-CoV-2 antigen has been reported in the postmortem pancreas of persons who died from COVID-19 [7]. Recently, both pancreatic islet autoantibodies and anti-S antibodies were detected in young children with a high genetic risk of T1D [8]. Conflicting results have been reported regarding the impact of SARS-CoV-2 infection on the occurrence of newly diagnosed T1D among children [9].

Infection can influence pancreatic *β*-cells by eliciting inflammation and promoting autoimmunity via molecular mimicry. Further, T cells activated in response to a microbial antigen also react with a self-antigen. The SARS-CoV-2 proliferates by binding to the angiotensin-converting enzyme 2 (ACE2) receptor on the surface of host epithelial cells. The SARS-CoV-2 virus has four structural proteins: the spike (S), nucleocapsid (N), membrane (M), and envelope (E). The S protein and its associated receptor-binding protein have been shown to interact with ACE2 in host cells, and this has been detailed as the first step of SARS-CoV-2 infection [10]. The SARS-CoV-2 N protein plays key roles in viral replication, assembly, pathogenesis, and antiviral immunity [11]. Its function is to integrate viral RNA into the ribonucleoprotein complex, which promotes the M and E proteins for viral assembly.

Serological tests are helpful for identifying asymptomatic and previously undiagnosed infections. Of particular importance are the neutralizing antibodies, which are capable of neutralizing the virus and, thus, provide protection against further infection. Several companies have developed diagnostic tools to test immunoglobulin G (IgG)-type antibodies that are directed against the S protein and antibodies directed against the N protein. Antibodies against the S protein are found both in people who were infected with SARS-CoV-2 and in people who were vaccinated with the BNT162b2 COVID-19 vaccine. In contrast, antibodies against the N protein are found only in people who had the SARS-CoV-2 infection. To detect the virus neutralizing antibodies, we developed a pseudoneutralizing assay based on a published protocol, as previously described [12]. We aimed to assess associations between seroprevalences of the different anti-SARS-CoV-2 antibodies and T1D occurrence in children and adolescents.

## 2. Methods

### 2.1. Study Design and the Population

This multicenter prospective observational cohort comprised two groups of children: children with T1D and a control group. The cohort included children and adolescents, aged 6 months to 18 years, who were diagnosed with T1D during the COVID-19 pandemic, between October 2020 and July 2022 in three central hospitals in Israel (Sheba Medical Center, Shamir Medical Center, and Tel Aviv Sourasky Medical Center). Institutional review board approval was granted by each institution, with Sheba Medical Center as the primary site, and informed consent was obtained. Each child with newly diagnosed diabetes was matched in a 1 : 3 ratio to unrelated children who were referred electively to perform endocrine tests in the outpatient clinic at Sheba Medical Center. The matching was according to sex, age, and the month of presentation at the hospital. Symptoms, anthropometric measurements, puberty status, biochemical findings (glucose, HbA1c and C-peptide levels, pancreatic antibodies), and diabetic ketoacidosis (DKA) status at T1D diagnosis were collected for the T1D group. The status of BNT162b2 mRNA COVID-19 vaccination was affirmed for both groups.

### 2.2. Serology Assays

All the samples were tested in the central virology laboratory of the Ministry of Health and in the endocrine laboratory located in Sheba Medical Center. The residual sera of all the children were tested for the presence of SARS-CoV-2 receptor-binding domain IgG antibodies (anti-S antibody). In the event that anti-S antibodies were found to be positive, anti-N antibodies and neutralizing antibodies were tested.

SARS-CoV-2 anti-S antibody analysis was performed using an in-house enzyme-linked immunosorbent assay (ELISA) based on the receptor-binding domain of the S protein [13]. Using a sample cutoff of 1.1, a lower value was defined as negative, and an equal or higher value was defined as positive. The test's sensitivity and specificity were 88% and 98%, respectively.

The SARS-CoV-2 pseudoneutralization assay was performed as described [12] to detect SARS-CoV-2 neutralizing antibodies using a green fluorescent protein reporter-based pseudotyped virus with a vesicular stomatitis virus backbone coated with the SARS-CoV-2 S protein.

SARS-CoV-2 N antibodies were measured using the Elecsys® Anti-SARS-CoV-2 electrochemiluminescence immunoassay (Cobas e801 Analyzer, Roche Diagnostics). The assay uses a recombinant protein representing the N antigen of SARS-CoV-2.

A positive result was defined as >1.0 cutoff index (COI). Accordingly, the magnitude of the measured result above the cutoff is not indicative of the total amount of antibodies present in the sample. The intra- and interassay coefficients of variation were 1.3%–2.9% and 2.1%–4.8%, respectively.

### 2.3. Statistical Analysis

Categorical variables were summarized as frequencies and percentages. Continuous variables were reported as medians and interquartile ranges. Chi-square tests and Fisher's exact tests were used to examine associations between categorical variables. Spearman's correlation was used to examine associations between continuous variables. The Mann–Whitney *U* test was applied to examine associations between continuous and categorical variables. All the statistical tests were two-sided, and *p* < 0.05 was considered as statistically significant. SPSS software was used for the statistical analysis (IBM SPSS Statistics for Windows, version 28.0, Armonk, NY, USA, 2021).

Scatter plots of neutralizing antibodies and mixed-model figures of log-transformed neutralizing antibodies (in log scale) of experimental groups were done using GraphPad Prism 9.0. The correlation between concentrations of log-transformed neutralizing antibodies was analyzed using Spearman's correlation with confidence intervals (CIs) of 95%. Log-transformed neutralizing antibody concentrations were analyzed as a continuous variable with multivariate linear regression.

## 3. Results

**Figure 1:**
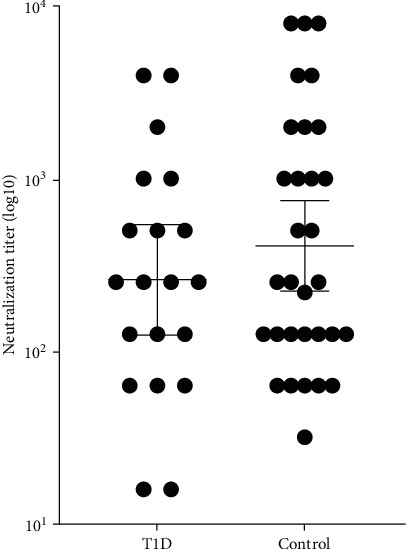
Quantification of neutralizing antibodies among unvaccinated children. Quantitation of neutralizing antibodies is shown among children who did not receive the BNT162b2 vaccine for children with type 1 diabetes (T1D) and matched controls. Neutralizing antibodies that were above the cutoff are presented. The dotted black line indicates the limit level of positive antibodies. The short black line indicates the geometric means and 95% CI.

The cohort included 51 children with T1D and 182 children in the control group.  Table 1 presents demographic characteristics and parameters related to COVID-19 for the two groups. The median (interquartile range (IQR)) age was 11.4 (IQR 8.2, 13.3) years, and 45% were female. The seroprevalences of the anti-S and the anti-N antibodies and the prevalence of neutralizing antibodies did not differ between the groups. The geometric mean titer of the neutralizing antibodies was half as much in the T1D group as in the control group (256 (IQR 64, 1,024) vs. 512 (IQR 128, 2,048), *p*=0.016). As the BNT162b2 COVID-19 vaccine can induce the neutralizing antibody titer [14], we adjusted for vaccination status. The resultant geometric mean titer did not differ between the groups (Table 1 and Figure 1).

Of the 51 children with T1D, 19 (37%) presented with DKA at diagnosis. The demographic, clinical, anthropometric, and laboratory characteristics according to DKA status at T1D presentation are shown in Table 2. Regarding COVID-19 seroprevalence, anti-S seroprevalence was higher among the non-DKA than the DKA group (72% vs. 42%, *p*=0.035); however, after adjustment to vaccination status, the difference was not statistically significant. Also, the seroprevalences of the anti-N antibodies and neutralizing antibodies did not differ between the groups. Among the children who presented with DKA, 6% were vaccinated, while among the children who did not present with DKA, 15% were vaccinated (*p*=0.3).

Associations were not found between anti-SARS-CoV-2 antibodies and glycemic parameters (HbA1c, C-peptide, or pancreatic antibody titer).

## 4. Discussion

We did not find an increase in the seroprevalence of any of the anti-SARS-CoV-2 antibodies among children diagnosed with T1D children between October 2020 and July 2022 compared to a control group. Among the T1D group, differences in the seroprevalence of the anti-S, anti-N, and the neutralizing antibodies were not statistically significant between the DKA group versus the non-DKA group. Furthermore, no associations were observed between any of the anti-SARS-CoV-2 antibodies and any of the glycemic parameters.

Evidence suggests that SARS-CoV-2 infection may harm pancreatic *β* cells, and whether or not this will lead to T1D depends on the balance between the balance between the loss of *β*-cell mass and the capacity of the remaining endogenous *β*-cells to regenerate [1]. Indeed, a review of the impact of the COVID-19 pandemic on the incidence of newly diagnosed T1D in children showed conflicting results [9].

Our findings corroborate those of a number of studies. Most of them did not assess COVID-19 seroprevalence, and documented instead either SARS-CoV-2 infection according to polymerase chain reaction (PCR) or only the incidence of new-onset T1D during the COVID-19 pandemic. A Finnish study [15] that analyzed SARS-CoV-2 antibodies among 75% of the children diagnosed with T1D reported that they were mostly negative. Out of 583 children examined in Finland, the percentage of children with a confirmed SARS-CoV-2 infection preceding the diagnosis of type 1 diabetes was less than 1% [15].

A Belgian study reported positive anti-SARS-CoV-2 antibodies in 20% of children diagnosed with T1D within the first month of diagnosis. This is similar to the proportion detected in the general population of children in Belgium [16]. In 2020 and 2021, Autoimmunity Screening for Kids (ASK) offered cross-sectional screening for both islet and SARS-CoV-2 antibodies to pediatric populations in Colorado, USA, and in Bavaria, Germany [17]. This project found that prior SARS-CoV-2 infection was not significantly associated with the presence of multiple islet autoantibodies or single high-affinity islet autoantibodies. Notably, none of the 465 Bavarian children who were followed with post SARS-CoV-2 infection developed islet autoantibodies. Other studies did not specifically estimate COVID-19 seroprevalences. Data from the Ministry of Health in Germany, from March 2020 to December 2021, showed no cross-correlations between COVID-19 incidence and T1D incidence for any age group [18]. Two studies from Canada [19, 20] and a study from Kuwait [21] found no differences in incidence rates of new T1D during the COVID-19 pandemic period compared to the prepandemic period.

A number of studies reported higher incidences of T1D during the COVID-19 pandemic period. Two multicenter studies from Germany found an increase in the incidence of new T1D, the first [22] during the first 18 months of the pandemic, and the second [23] covered the years 2020–2021, and compared the incidence during those years to the period of 2011–2019.

A study from UK [24] evaluated the incidence of new T1D in five centers during a short period—the first lockdown—from March to May 2020, a period different from that of our study. In two of five centers, there was an apparent increase, but not in the other three. One single tertiary center in the USA published a 1.5-fold increase in the incidence of newly diagnosed T1D during the first year of the pandemic [25]. Similarly, a Brazilian study found a twofold increase in the first half year of the pandemic [26] compared to prior years. The Centers for Disease Control and Prevention (CDC) reported that during the first year of the pandemic, the incidence of any kind of diabetes was 1.3–2.6-fold higher among individuals with COVID-19 than among those without COVID-19. The incidence of diabetes was also 2.2-fold higher than the incidence among those with non-COVID-19 acute respiratory infection in the prepandemic period [27]. A study utilizing the Global Collaborative Network, of over one million pediatric patients, compared those who were infected with SARS-CoV-2 between March 2020 and December 2021 and those who were not infected but contracted another respiratory infection during the same period [28]. Increased rates of T1D diagnoses were shown at 1, 3, and 6 months after SARS-CoV-2 infection compared to non-COVID-19 respiratory infections. Last, two meta-analysies of children with T1D showed a higher incidence rate ratio during the first year of the pandemic compared with the prepandemic period [29, 30]. It is possible that the acute illness following COVID-19 unveiled and triggered the onset of T1D in individuals already predisposed to the condition.

Despite the controversy in the literature regarding the impact of SARS-CoV-2 infection and T1D incidence in the pediatric population, the increases in DKA frequency and severity are undeniable [9, 20, 31–38]. However, this increase was attributed to referral delay during the lockdown period and not to the increased incidence of T1D [20, 31, 32, 34].

Among the T1D group, once adjusted to vaccination status, the difference in anti-S antibody seroprevalence disappeared. In addition, the anti-N seroprevalence, which indicates acute SARS-CoV-2 infection, did not differ between the groups.

The pseudoneutralizing SARS-CoV-2 antibody assay enables performing neutralizing assays in a Biosafety Level 2 lab and avoiding the dangers of the real COVID-19 virus [39]. These assays have been shown to correlate with protection; thus, their characterization is vital to tracking immunogenicity. However, as the assays were shown to be affected after BNT162b2 COVID-19 vaccine injection [14], the assay titer must be adjusted to vaccination status to prolong host immunity [14]. After adjusting for vaccination status in our cohort, no differences were observed in the assay titer. This reflects a similar protection from the SARS-CoV-2 infection both in the control group and in the T1D group.

Our research has some limitations. First, information was lacking regarding the various COVID-19 variants. This may have affected the association between the occurrence of T1D and the SARS-CoV-2 infection. Second, three hospitals in the center of Israel were included and not centers from other parts of the country. Third, the control group was only from one medical center; however, it is important to note that there was no difference in the seroprevalences of anti-SARS-CoV-2 antibodies between the three different medical centers, suggesting that there was no bias. Furthermore, the children in the control group were outpatients and not hospitalized children. Last, our cohort was not large, consisting of 51 children with T1D and 182 children in the control group. Eight out of 51 (16%) children with T1D were under the age of 6 years, an age group with a higher rate of T1D incidence during the COVID-19 pandemic. However, our trial had a lower rate of young children diagnosed with T1D compared to an international study in which 25% were under the age of 6 years [31].

A noteworthy aspect of our research is that we measured anti-N and neutralizing antibodies, essentially distinct anti-SARS-CoV-2 antibodies. This contrasts with previous studies on children with T1D, which examined only anti-S antibodies. In addition, we assessed various titers, particularly those of neutralizing antibodies that provide COVID-19 protection and observed no distinctions between children with T1D and the control group. Finally, our study focused on antibodies rather than acute PCR. Anti-S and anti-N antibodies were also measured in a trial from the UK involving a healthy population and were shown to be in similar titers irrespective of age [40]. Anti-N and neutralizing antibodies were also checked in a Finnish study but only in nine out of 583 children who were positive for anti-S antibodies and without comparing them to a control group [15].

In summary, we did not find any association between the occurrence of T1D and SARS-CoV-2 infection. The rate of SARS-CoV-2 infection, as documented by anti-N antibodies, was not higher among children with than without DKA. Since autoimmunity may emerge years after a viral infection, we recommend conducting follow-up epidemiological studies to assess whether there is a change in the incidence of T1D following the SARS-CoV-2 pandemic.

## Figures and Tables

**Table 1:** COVID-19 seroprevalence of children with type 1 diabetes and matched controls.

Characteristic	Control (*n* = 182)	Diabetes (*n* = 51)	*p*-value
Age (years)	11.4 (8.3–13.3)	11.1 (7.5–13.6)	0.9
Female gender *n* (%)	84 (46.2)	23 (45.1)	0.9
Positive anti-S antibodies *n* (%)	102 (56)	30 (59)	0.7
Positive anti-N antibodies *n* (%)	65 (36)	19 (37)	0.7
Positive neutralizing antibodies *n* (%)	101 (55)	28 (55)	0.2
Neutralizing antibody value, unadjusted to vaccination status	512 (128–2,048)	256 (64–1,024)	0.02
Neutralizing antibody value, adjusted to vaccination status	256 (80–896)	256 (128–2,048)	0.5

All the parameters are displayed as median (Q1, Q3) except the neutralizing antibodies, which are displayed as geometric means.

**Table 2:** Characteristics of children with type 1 diabetes according to diabetic ketoacidosis (DKA) status at diagnosis.

Parameter category	Parameter	DKA (*n* = 19)	Non-DKA (*n* = 32)	*p*-value
Demographic and anthropometric parameters	Age (years)	9.7 (6.7–12.7)	11.5 (9.0–14.2)	0.2
	Female gender	9 (47%)	14 (44%)	0.8
	Weight SDS	0.16 (−0.53 ± 0.81)	−0.38 (−0.9 ± 0.31)	0.09
	Height SDS	0.38 (+0.05 ± 0.78)	1.42 (1.27 ± 1.6)	0.2
	BMI SDS	0.44 (−1.2 ± 1.07)	−0.02 (−1.96 ± 0.46)	0.2
	Puberty	8 (42%)	12 (38%)	0.2

Clinical parameters	Polyuria	19 (100%)	29 (91%)	0.18
	Polydipsia	19 (100%)	27 (84%)	0.068
	Gastrointestinal symptoms	10 (53%)	9 (28%)	0.07
	Weight loss	16 (84%)	16 (50%)	0.016
	Weakness	17 (89%)	17 (53%)	0.009

Laboratory parameters	pH	7.14 (7.09–7.21)	7.36 (7.32–7.39)	<0.001
	HCO_3_^−^ (mmol/l)	7.9 (6.6–10.0)	21 (18–24)	<0.001
	Glucose (mg/dL)	589 (445–678)	455 (318–555)	0.02
	Glucose (mmol/l)	32.7 (24.7–37.7)	25.3 (17.7–30.8)	0.02
	HbA1c (%)	11.8 (10.9–12.7)	11.8 (9.7–14.9)	0.8
	HbA1c (mmol/mol)	105 (96–115)	105 (83–139)	0.8
	C-peptide (*μ*g/l)	0.14 (0.23–0.27)	0.27 (0.42–0.57)	0.036
	Anti-GAD titer (IU/ml)	51.6 (6.2–512)	44 (4.9–176)	0.4
	Anti-IA2 titer (IU/ml)	17 (7.4–351)	7.1 (2.8–218)	0.065
	Anti-insulin titer	0.38 (0.30–0.49)	0.37 (0.3–0.8)	0.9
	Positive anti-S antibodies	8 (42%)	23 (72%)	0.035
	Positive anti-S antibodies, adjusted to vaccination status	15/26 (58%)	8/20 (40%)	0.7
	Positive anti-N antibodies	7 (37%)	14 (44%)	0.2
	Positive neutralizing antibodies	9 (47%)	22 (69%)	0.1
	Neutralizing antibody value, unadjusted to vaccination status	96 (64–512)	256 (64–2,048)	0.8
	Neutralizing antibody value, adjusted to vaccination status	512 (64–448)	256 (128–1,024)	0.4

Categorical variables are introduced as *n* (%) and continuous variables as medians (Q1, Q3). Neutralizing antibodies are displayed as geometric mean.

## Data Availability

Data will be released upon request.
